# Effects of Dietary Supplementation of Algae-Derived Polysaccharides on Morphology, Tight Junctions, Antioxidant Capacity and Immune Response of Duodenum in Broilers under Heat Stress

**DOI:** 10.3390/ani11082279

**Published:** 2021-08-02

**Authors:** Wen-Chao Liu, Yan-Ru Zhu, Zhi-Hui Zhao, Ping Jiang, Fu-Quan Yin

**Affiliations:** Department of Animal Science, College of Coastal Agricultural Sciences, Guangdong Ocean University, Zhanjiang 524088, China; liuwc@gdou.edu.cn (W.-C.L.); zhuyanru1@stu.gdou.edu.cn (Y.-R.Z.); zhzhao@gdou.edu.cn (Z.-H.Z.)

**Keywords:** broilers, seaweed polysaccharides, heat stress, intestinal health, signaling pathway

## Abstract

**Simple Summary:**

Heat stress (HS) has become a great challenge for poultry production in tropical and subtropical regions. HS results in the intestinal dysfunction of broilers, which seriously affects their productivity. Our previous study suggested that dietary supplementation of algae-derived polysaccharides (ADP) could promote the intestinal barrier function in broilers, but the effect of dietary ADP supplementation on the intestinal health of broilers under HS remains unclear. The present study showed that dietary ADP supplementation improved the duodenal tight junction expression of broilers under HS, and found that dietary ADP mitigated HS-induced oxidative stress and inflammation response by regulating Nrf2 and NF-κB signaling pathways. These findings reveal the potential application of ADP as an HS-alleviating agent to maintain gut health in broilers.

**Abstract:**

To evaluate the ameliorative effect of algae-derived polysaccharide (ADP) supplementation on duodenal injury caused by heat stress (HS) in broilers, a total of 144 male yellow-feathered broilers (56-day-old) were randomly allocated into three groups: The TN group (thermoneutral zone, broilers were raised at 23.6 ± 1.8 °C); HS group (heat stress, broilers were exposed to 33.2 ± 1.5 °C 10 h/day, 8:00 a.m.–18:00 p.m., the temperature in the remaining period was consistent with the TN group); HSA group (heat-stressed broilers were fed with ADP supplemented diet at 1000 mg/kg). There were six replications in each treatment, and eight broilers in each replication. The feeding trial lasted four weeks. The results showed that dietary ADP supplementation tended to increase the villus height (*p* = 0.077) and villus width (*p* = 0.062), and decrease the apoptosis rate (*p* = 0.081) in the duodenum of broilers under HS. Furthermore, dietary ADP increased the relative mRNA and protein (based on immunofluorescence) expression levels of occludin and zonula occludens-1 (ZO-1) in the duodenum of broilers under HS (*p* < 0.05). In addition, dietary ADP enhanced the total antioxidation capacity (T-AOC) and activity of glutathione-S transferase (GST), while reducing the malondialdehyde (MDA) concentration of the duodenum in broilers under HS (*p* < 0.05). Moreover, dietary ADP supplementation upregulated the duodenal nuclear factor erythroid 2-related factor 2 (*Nrf2*), heme oxygenase-1 (*HO-1*), glutathione peroxidase 1 (*GPx1*) and glutathione S-transferase theta 1 (*GSTT1*) mRNA expression levels in heat-stressed broilers (*p* < 0.05). Furthermore, compared with the HS group, broilers fed with an ADP supplemented diet had a higher relative mRNA expression of inhibitor kappa B alpha (*IκBα*) (*p* < 0.05) and a lower relative mRNA expression of tumor necrosis factor-α (*TNF-α*) and interleukin-1β (*IL-1β*) in the duodenum (*p* < 0.05). In summary, dietary ADP supplementation had an ameliorative effect on HS-induced impairment of tight junctions, antioxidant capacity and the immune response of the duodenum in broilers. These beneficial effects might be related to the modulation of Nrf2 and NF-κB signaling pathways.

## 1. Introduction

Heat stress (HS) is a great challenge for broiler production in tropical and subtropical regions [1]. HS results in a reduction of feed consumption, degradation of growth rate, abnormalities of metabolism, imbalance of electrolytes, and disorders of the endocrine system in broilers [2,3,4,5,6]. As one of the most important digestive organs, the gut is not only the place for digestion and absorption of nutrients, but also plays a crucial role in barrier function, and it is highly sensitive to HS [7]. HS exposure causes intestinal dysfunction, impairs gut barrier integrity and induces an intestinal oxidative stress and inflammation response in chickens [8,9,10,11]. Therefore, using a protective approach against HS-induced impairment of intestinal health is important for broiler production. It is currently believed that nutritional regulation is an effective strategy to protect the broilers against HS-induced intestinal injury [12,13]. In this regard, a variety of natural phytochemicals were used as gut health promoters in broilers, and they showed potential stress-alleviating effects because of the multiple biological activities [14,15,16].

Polysaccharides, as natural active polymers, are widely present in plants and microorganisms. In recent years, polysaccharides have attracted great attention in investigating alternative growth promoters in animals [17]. For instance, numerous studies observed that dietary medicinal plant-derived polysaccharides could enhance immune function [18], elevate antioxidant capacity [19], improve intestinal barrier function [20,21,22], thus promoting the growth performance of broilers. In addition, previous studies found that dietary natural polysaccharides extracted from *Atractylodes* could mitigate HS-induced multiple organ dysfunction of broilers by suppressing apoptosis and oxidative stress [23,24]. Marine polysaccharides have received extensive attention because of their biological activities [25,26]. *Enteromorpha prolifera* is a kind of wild marine green algae, which belongs to the *Ulvaceae* family and *Enteromorpha* genus. It is widely distributed in various offshore areas. *E. prolifera* has good edible and medicinal values, showing anti-inflammatory and detoxifying effects [27]. *E. prolifera* contains abundant polysaccharides that exhibit many biological properties, including antioxidant, antiviral, antitumor, anti-inflammatory, immunomodulatory and antibacterial properties [28,29]. In our previous studies, the dietary inclusion of algae (*E. prolifera*)-derived polysaccharides (ADP) improved the intestinal barrier function of laying hens [30] and broilers [31]. Furthermore, dietary ADP supplementation showed mitigating effects on oxidative stress and apoptosis in broilers under HS or after aflatoxin B_1_ exposure [32,33]. However, little is known about the effects of dietary ADP supplementation on the intestinal health of broilers under HS conditions. Therefore, the present study aimed to evaluate the ameliorative effect of ADP supplementation on HS-induced duodenal injury of broilers and explore the underlying molecular mechanism.

## 2. Materials and Methods

### 2.1. Birds, Diet, and Trial Design

One hundred and forty-four 56-day-old male Chinese indigenous, yellow-feathered broilers were used for a four-week feeding trial. The broilers were purchased from a local chicken hatchery (Maoming, Guangdong, China) The age selection was based on the fact that broilers are more sensitive to HS during the growth and finishing period, and 56–84 days are known as the growing−finishing stages for Chinese indigenous, yellow-feathered broilers [6]. Broilers were randomly assigned to three groups: TN group (thermoneutral zone, broilers were raised at 23.6 ± 1.8 °C); HS group (heat stress, broilers were exposed to 33.2 ± 1.5 °C 10 h/day, 8:00 a.m.–18:00 p.m., the temperature in the remaining period was consistent with the TN group); HSA group (heat-stressed broilers were fed with ADP at 1000 mg/kg). The relative humidity of all groups was maintained at 55–75%. There were six replications and eight broilers/replication in each treatment. The temperature and relative humidity of three different locations in the chicken house were detected six times per day, and the final temperature and relative humidity were the average data recorded daily. The initial body weight (average initial body weight, 682.59 ± 7.38 g) between the three treatments had no significant difference. The chickens were fed with corn-soybean meal-based diet, and the feed formulation was the same as described in our previous literature [33]. The ADPs were enzymatically extracted from *E. prolifera*, and the detailed information of the ADP was mentioned in our previously studies [30,31,33]. Briefly, the ADPs are water-soluble sulfated polysaccharides (purity ≥ 48%). The composition of monosaccharide is rhamnose (Rha, 40.6%), glucose (Glc, 38.2%), glucuronic acid (GlcA, 9.3%), xylose (Xyl, 6.3%) and galactose (Gal, 5.6%).

### 2.2. Collection of Duodenal Samples

At the end of the feeding experiment, one broiler from each replicate was randomly selected and euthanized, and then the duodenal segments were separated. About 2 cm segment of the duodenal middle section was carefully collected and put into 4% paraformaldehyde for morphology, apoptosis and immunofluorescence analyses (*n* = 6 per treatment). Subsequently, the remaining segments of the duodenum were cut. Then, the digesta was rinsed with phosphate-buffered (PBS) solution at 4 °C, and then the duodenal mucosal samples were scraped with a glass slide (*n* = 6 per treatment). All duodenal mucosal samples were put into liquid nitrogen, transported to the laboratory and stored at −80 °C for antioxidant and molecular analyses.

### 2.3. Determination of Morphology and Apoptosis in Duodenum

The detection and analysis of duodenal morphology were based on our previous studies [6,33]. Briefly, the duodenal segments were fixed in 4% paraformaldehyde for 48 h, then the duodenal segments were embedded in paraffin and cut into 5 μm sections, and stained with hematoxylin and eosin (H & E). The section figures and the villus height, villus width and crypt depth of the duodenum were collected and measured using TCapture Imaging Application 4.3 software, then villus height/crypt depth ratio was calculated.

The apoptosis of duodenal epithelial cells was detected and analyzed by paraffin section TdT-mediated dUTP Nick-End Labeling (TUNEL) method. The assay kit and detection details as described previously [33]. Briefly, add the TdT and dUTP reagents from TUNEL kit into the slices to cover the duodenal samples, then incubate at 37 °C for 2 h, and wash the slices with PBS (pH 7.4). After removing the PBS, drip 4′,6-diamidino-2-phenylindole (DAPI) staining solution on the slices, and incubate at room temperature for 10 min. Finally, TUNEL slices were observed and analyzed under 100× magnification using Image-Pro Plus 6.0 software (Media Cybernetics, Inc., Rockville, MD, USA). The apoptosis rate was calculated as per the following formula:

Apoptosis rate (%) = (positive apoptotic cells/total cells) * 100%

### 2.4. Barrier Function-Related Molecular Analysis

The mRNA expression levels of intestinal barrier function-related genes (*Cadherin*, *Occludin*, *Claudin-1*, *Claudin-4*, *ZO-1* and *Mucin-2*) were examined by quantitative real-time PCR (qRT-PCR). The total RNA extraction of duodenal mucosal samples was done. qRT-PCR reaction conditions and operation details were the same as described in our former studies [32,33]. Briefly, the qPCR reaction was performed using the CFX-96 real-time PCR detection system (BioRad, Irvine, CA, USA). The total reaction volume was 20 μL. The PCR program was 95 °C for 30 s, and 40 cycles of 95 °C for 10 s, 60 °C for 30 s and 72 °C for 15 s. The internal reference gene was *β-actin*, and the details of the primers were presented in our earlier study [9].

The immunofluorescence of duodenum was conducted to detect the expression of selected barrier function-related molecules. The selection of molecules was based on the qRT-PCR results. The immunofluorescence detection and analysis information were given as follows: firstly, the diluted fluorescently labeled (green) target protein primary antibodies (rabbit polyclonal antibody against occludin: Servicebio, Wuhan, China, GB111401, dilution ratio: 1:200; rabbit polyclonal antibody against ZO-1 (zonula occludens-1): Proteintech, Wuhan, China, 21773-1-AP, dilution ratio: 1:100) were added into the duodenal sections, incubated overnight at 4 °C and rinsed with PBS. Then, the diluted secondary antibodies (goat polyclonal secondary antibody to rabbit IgG, Alexa Fluor 488, Servicebio, Wuhan, China, GB25303, dilution ratio: 1:400) were added into the duodenal sections, and the DAPI was used to label the cell nucleus, then incubated at room temperature for 1 h in the dark and rinsed with PBS. Upright fluorescence microscope (Nikon Eclipse, C1, Japan) was used to observe the sections and collect images (Nikon, DS-U3). Image-Pro Plus 6.0 (Media Cybernetics, Inc., Rockville, MD, USA) software was used to analyze and calculate the areal density (AD) value. The analysis method was presented as follows: three fields of view for each slice were selected to take photos under 100× magnification for converting the fluorescent monochrome photos into black and white images using Image-Pro Plus 6.0 software. The same black image was selected as the unified standard, and then the integrated optical density (IOD) of positive expression and the pixel area of the tissue (AREA) for each positive photo was obtained. The AD value was calculated as follows: AD = IOD/AREA. The AD value indicates the expression level of the target protein.

### 2.5. Antioxidant Capacity Analysis

The antioxidant parameters of duodenal mucosal samples, including total anti-oxidation capacity (T-AOC), activities of the total superoxide dismutase (T-SOD), glutathione peroxidase (GSH-Px), catalase (CAT), glutathione-S transferase (GST), and malondialdehyde (MDA) levels, which were detected according to our earlier study [33].

The relative mRNA expression of antioxidant signaling pathway-related genes, including *Nrf2* (nuclear factor erythroid 2-related factor 2), *HO-1* (heme oxygenase-1), *SOD1* (superoxide dismutase-1), *SOD2* (superoxide dismutase-1), *GPx1* (glutathione peroxidase-1), *GPx3* (glutathione peroxidase-3), *CAT1* (catalase-1), *GSTT1* (glutathione S-transferase theta-1) and *GSTO1* (glutathione S-transferase omega-1) were analyzed using qRT-PCR. The qRT-PCR conditions were the same as described previously [32,33]. The internal reference gene was *β-actin*, the details of primers were described in our previous study [33].

### 2.6. Immune Response Analysis

To analyze the immune response of duodenal mucosal samples, mRNA expression of immune-related signaling pathway genes was performed, using qRT-PCR and the detection method as described previously [32,33]. *β-actin* was used as the internal reference gene, the primer information was presented in our former report [33]. The genes contained *IκBα* (inhibitor kappa B alpha), *NF-κB p65* (nuclear factor-kappa B p65), *TNF-α* (tumor necrosis factor-α), *IFN-γ* (interferon-γ), *IL-1β* (interleukin-1β), *IL-2* (interleukin-2), *IL-4* (interleukin-4), *IL-6* (interleukin-6) and *IL-10* (interleukin-10).

### 2.7. Statistical Analysis

The mixed model procedure was used to analyze the data by SAS 9.4 (SAS, 2013. SAS Institute Inc., Cary, NC, USA). The model was used as follows: *Y_ijk_ = μ + t_i_ + r_k_ + e_ijk_*, where *Y_ijk_* is an observation on the dependent variable *ij*, *μ* is the overall population mean, *t_i_* is the fixed effect of treatments, *r_k_* is the individual bird as a random effect, and *e_ijk_* is the random error associated with the observation *ijk*. The individual bird as the experimental unit. Data were expressed as means. Tukey’s test (multiple comparison) was performed to analysis the differences between treatments. *p* < 0.05 was considered to be statistically significant, and 0.05 ≤ *p* < 0.10 was considered to be a significant trend.

## 3. Results

### 3.1. Duodenal Morphology and Apoptosis

**Figure 1:**
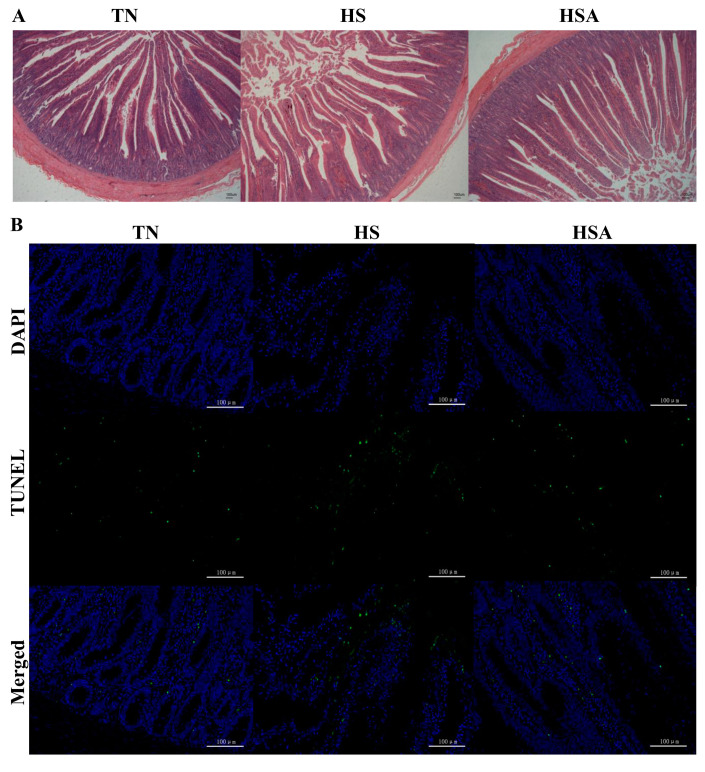
Duodenal sections showing the influence of algae-derived polysaccharides (ADP) on morphology (**A**) and cell apoptosis (**B**) in heat-stressed broilers. Note: TN group (thermoneutral zone, broilers exposed to 23.6 ± 1.8 °C); HS group (heat-stress, broilers exposed to 33.2 ± 1.5 °C for 10 h/day); HSA group (heat-stressed broilers were fed with ADP at 1000 mg/kg); TUNEL, TdT-mediated dUTP Nick-End Labeling; DAPI, 4′,6-diamidino-2-phenylindole. The scale bar for morphology (**A**, 40×) and cell apoptosis (**B**, 100×) is 100 μm.

**Figure 2:**
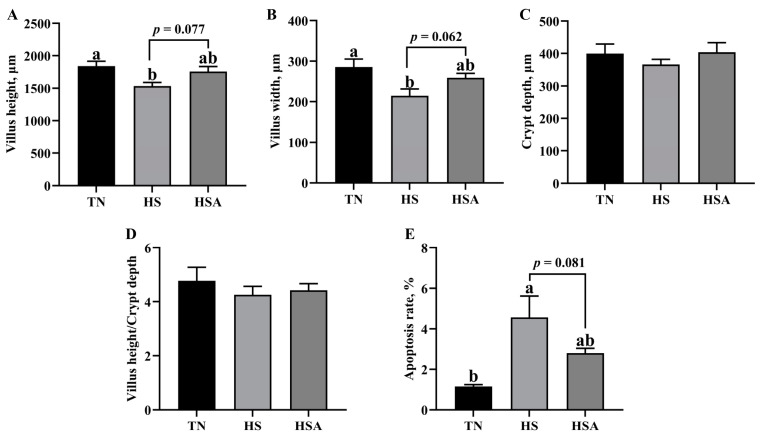
Influence of algae-derived polysaccharide (ADP) supplementation on villus height (**A**), villus width (**B**), crypt depth (**C**), villus height/crypt depth (**D**) and apoptosis rate (**E**) in heat-stressed broilers. Note: TN group (thermoneutral zone, broilers exposed to 23.6 ± 1.8 °C); HS group (heat stress, broilers exposed to 33.2 ± 1.5 °C for 10 h/day); HSA group (heat-stressed broilers were fed with ADP at 1000 mg/kg). The data were expressed as means ± standard error (*n* = 6). ^a,b^ The significant differences between groups were expressed as different superscript letters in the Figures (*p* < 0.05).

As shown in Figure 1 and Figure 2, HS reduced the duodenal villus height and villus width (*p* < 0.05), while it increased the duodenal apoptosis rate (*p* < 0.05). In addition, ADP supplementation tended to improve the duodenal villus height and villus width (*p* < 0.10), and decrease the duodenal apoptosis rate in heat-stressed broilers (*p* < 0.10).

### 3.2. Duodenal Barrier Function Related Molecular Expression

**Figure 3:**
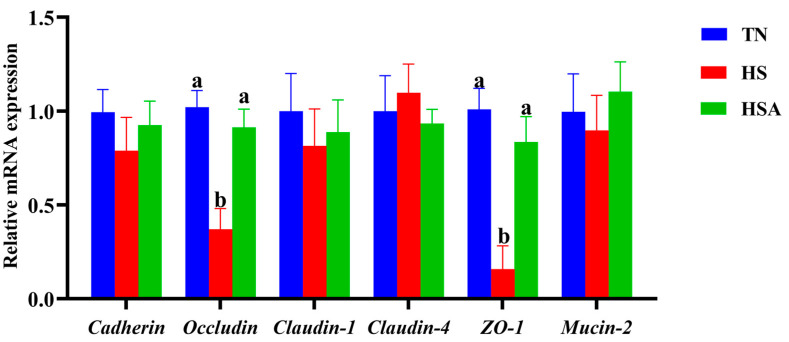
Influence of algae-derived polysaccharide (ADP) supplementation on relative mRNA expression of duodenal barrier function related genes in heat-stressed broilers. Note: TN group (thermoneutral zone, broilers exposed to 23.6 ± 1.8 °C); HS group (heat stress, broilers exposed to 33.2 ± 1.5 °C for 10 h/day); HSA group (heat-stressed broilers were fed with ADP at 1000 mg/kg). The data were expressed as means ± standard error (*n* = 6). ^a^^,b^ The significant differences between groups were expressed as different superscript letters in the Figures (*p* < 0.05).

**Figure 4:**
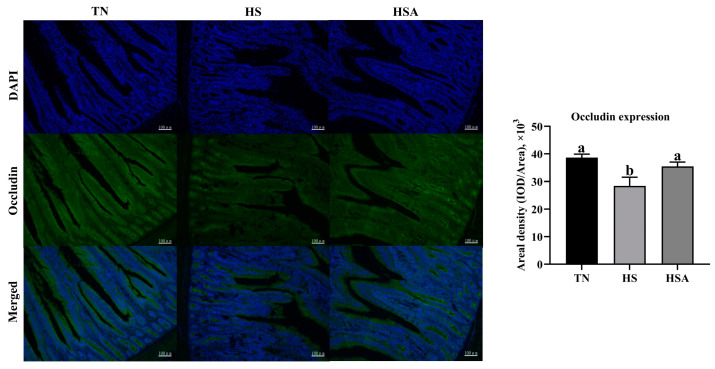
Influence of algae-derived polysaccharide (ADP) supplementation on duodenal occludin expression based on immunofluorescence in heat-stressed broilers. Note: TN group (thermoneutral zone, broilers exposed to 23.6 ± 1.8 °C); HS group (heat stress, broilers exposed to 33.2 ± 1.5 °C for 10 h/day); HSA group (heat-stressed broilers were fed with ADP at 1000 mg/kg). The data were expressed as means ± standard error (*n* = 6). ^a,b^ The significant differences between groups were expressed as different superscript letters in the Figures (*p* < 0.05).

**Figure 5:**
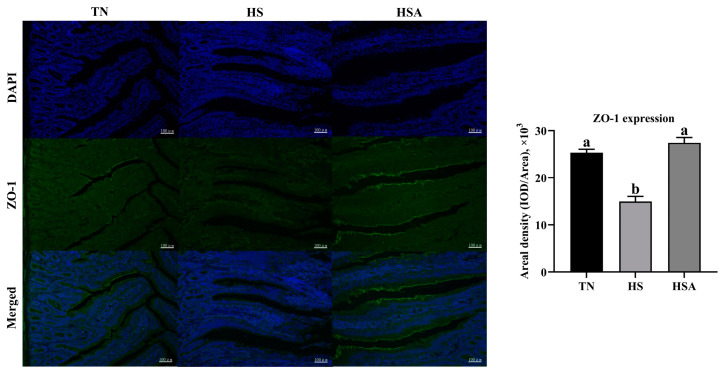
Influence of algae-derived polysaccharide (ADP) supplementation on duodenal ZO-1 expression based on immunofluorescence in heat-stressed broilers. Note: TN group (thermoneutral zone, broilers exposed to 23.6 ± 1.8 °C); HS group (heat stress, broilers exposed to 33.2 ± 1.5 °C for 10 h/day); HSA group (heat-stressed broilers were fed with ADP at 1000 mg/kg). The data were expressed as means ± standard error (*n* = 6). ^a,b^ The significant differences between groups were expressed as different superscript letters in the Figures (*p* < 0.05).

As illustrated in Figure 3, compared with the TN group, a lower relative mRNA expression of *occludin* and *ZO-1* of the duodenum was observed in HS group (*p* < 0.05). Compared with the HS group, a higher relative mRNA expression of *occludin* and *ZO-1* of the duodenum was observed in HSA group (*p* < 0.05). According the results of immunofluorescence (Figure 4 and Figure 5), HS exposure reduced duodenal occludin and ZO-1 expression (*p* < 0.05), and ADP supplementation improved the duodenal occludin and ZO-1 expression in broilers under HS (*p* < 0.05).

### 3.3. Antioxidant Capacity of Duodenum

The results of the antioxidant enzyme activities and the MDA content of the duodenum are presented in Table 1. HS exposure decreased the T-AOC and activity of GST, but increased the MDA levels of the duodenum (*p* < 0.05). Dietary ADP improved the T-AOC and activity of GST, while reducing the MDA concentration of the duodenum in heat-stressed broilers (*p* < 0.05).

**Figure 6:**
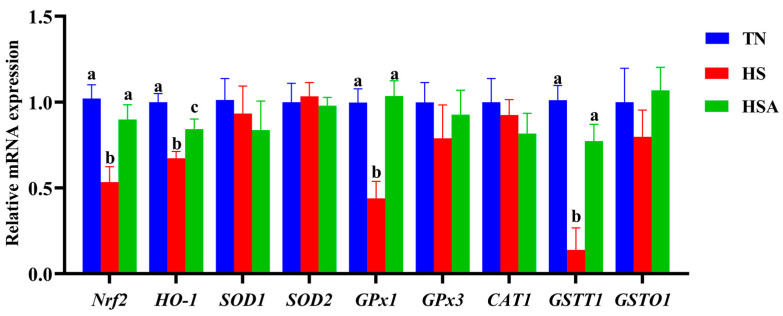
Influence of algae-derived polysaccharide (ADP) supplementation on duodenal relative mRNA expression of antioxidant signaling-pathway-related genes in heat-stressed broilers. Note: TN group (thermoneutral zone, broilers exposed to 23.6 ± 1.8 °C); HS group (heat stress, broilers exposed to 33.2 ± 1.5 °C for 10 h/day); HSA group (heat-stressed broilers were fed with ADP at 1000 mg/kg). The data were expressed as means ± standard error (*n* = 6). ^a,b,c^ The significant differences between groups were expressed as different superscript letters in the Figures (*p* < 0.05).

As shown in Figure 6, the relative mRNA expression of *Nrf2*, *HO-1*, *GPx1* and *GSTT1* of the duodenum were downregulated by HS exposure (*p* < 0.05). However, the duodenal relative mRNA expression of *Nrf2*, *HO-1*, *GPx1* and *GSTT1* in heat-stressed broilers was upregulated by ADP supplementation (*p* < 0.05).

### 3.4. Immune Response of Duodenum

**Figure 7:**
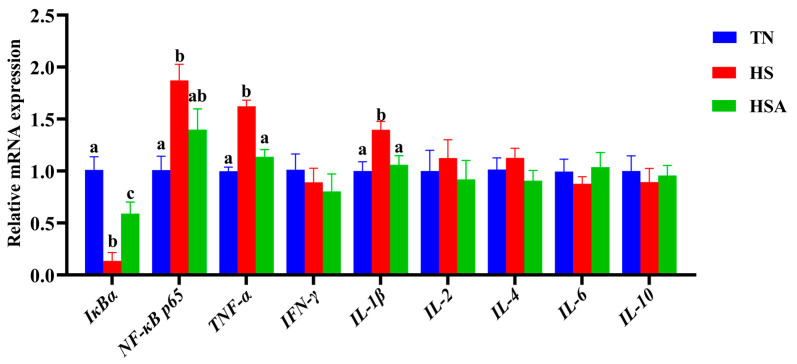
Influence of algae-derived polysaccharide (ADP) supplementation on duodenal relative mRNA expression of immune signaling-pathway-related genes in heat-stressed broilers. Note: TN group (thermoneutral zone, broilers exposed to 23.6 ± 1.8 °C); HS group (heat stress, broilers exposed to 33.2 ± 1.5 °C for 10 h/day); HSA group (heat-stressed broilers were fed with ADP at 1000 mg/kg). The data were expressed as means ± standard error (*n* = 6). ^a,b^^,c^ The significant differences between groups were expressed as different superscript letters in the Figures (*p* < 0.05).

As presented in Figure 7, the duodenal relative mRNA expression of *IκBα* was downregulated by HS (*p* < 0.05), while the duodenal relative mRNA expression of *NF-κB p65*, *TNF-α,* and *IL-1β* was upregulated by HS (*p* < 0.05). In addition, dietary ADP upregulated the duodenal relative mRNA expression of *IκBα* (*p* < 0.05) and downregulated the duodenal relative mRNA expression of *TNF-α,* and *IL-1β* (*p* < 0.05). There were no significant differences in the duodenal relative mRNA expression of *NF-κB p65* between TN and HSA groups (*p* > 0.10).

## 4. Discussion

### 4.1. Duodenal Morphology and Apoptosis

The broilers often suffer from a variety of stressors, and the gut health of broilers is severely influenced by HS due to global warming and high-density farming [7,34]. In this study, the results showed that a four-week HS exposure reduced the duodenal villus height and width, and increased the epithelial cell apoptosis of the duodenum in yellow-feathered broilers. Consistently, Liu et al. [6] found that 5 weeks of HS decreased the duodenal villus height of yellow-feathered broilers. He et al. [35] suggested that 14 d of HS exposure induced a reduction in jejunal villus height of yellow-feathered broilers. Zhang et al. [14] reported that the Cobb broilers subjected to 21 d of HS resulted in a lower villus height and ratio of villus height to crypt depth. He et al. [36] also demonstrated that 14 d heat exposure increased the apoptosis cells in the jejunal villus of male Arbor Acres broilers. The peripheral blood flow of broilers increased to accelerate heat dissipation, causing insufficient blood supply to the digestive tract and resulting in intestinal epithelial cell injury under HS [10], which may be the reason for the impairment of morphology and apoptosis in the duodenal epithelial cells by HS. In recent years, numerous studies have tried to alleviate the intestinal morphological damage of heat-stressed broilers by natural polysaccharide dietary supplementation, which has been suggested to exert beneficial effects [20,21,37]. Meanwhile, our previous studies demonstrated that the ADP could act as a gut health promoter and improve the small intestinal villus height in Arbor Acres broilers [31] and late-phase laying hens [30] under thermoneutral conditions. However, the present study only showed a trend of a mitigation effect on morphological injury and apoptosis rate in the duodenum of heat-stressed broilers fed with an ADP-supplemented diet. These inconsistencies are probably due to the HS duration, broiler breeds and polysaccharide types.

### 4.2. Tight Junctions in Duodenum

The physical intestinal barrier plays an important role in preventing harmful bacteria and endotoxins from entering the blood circulation [38]. The junction complex composed of tight junctions, adhesion junctions and desmosome between adjacent cells of the intestinal epithelium is the main physical barrier. The physical intestinal barrier-related molecules include cadherin, occludin, claudins, ZOs, mucins, etc., [7,39]. In this study, based on the analysis of selected physical gut barrier function-related molecular expression, four-week HS exposure decreased the tight junction protein expression of the duodenum, including occludin and ZO-1, and their absence could be responsible for an increase in intestinal permeability. Similar findings were also obtained by Liu et al. [9] and He et al. [35]. Meanwhile, the present study evaluated the mitigation effects of dietary ADP supplementation on duodenal barrier function-related molecular expression of broilers under HS. As expected, the dietary inclusion of ADP improved the tight junction (occludin and ZO-1) expression levels in the duodenum of broilers under HS. In agreement, Sandner et al. [40] demonstrated that dietary ginseng extract (enriched polysaccharides) improved the *occludin* expression of the jejunum in Cobb broilers under HS conditions (d 22–42). According to Tellez [41], dietary *Morinda Citrifolia* polysaccharides could upregulate the expression levels of *ZO-1* and *ZO-2* genes in the small intestine of Cobb broilers exposed by HS for one week.

### 4.3. Antioxidant Capacity in the Duodenum

Improving the antioxidant capacity can maintain the redox balance and normal physiological functions of intestinal epithelial cells. According to the available literature, HS has a negative influence on the antioxidant capacity and leads to oxidative stress, which can damage the cells and tissues in broilers [10,12,13]. Meanwhile, the Nrf2-mediated antioxidant signaling pathway plays a key role in mitigating oxidative stress [42]. As an important cytoprotective transcription factor, Nrf2 is associated with the promotion of antioxidant capacity by regulating phase II detoxifying and antioxidant enzyme expression, such as HO-1, GSH-Px, CAT, GST, and SOD [43]. Nrf2 overexpression and nuclear translocation can promote the expression of downstream antioxidant genes, thereby improving the antioxidant capacity [44]. It has been suggested that broilers under HS conditions showed lower antioxidant capacity, which is related to the suppression of Nrf2 expression in broilers [33,45,46,47]. This study found that HS exposure decreased the antioxidant enzyme activities and elevated the MDA content in the duodenal mucosa, which is a biomarker of lipid peroxidation. Furthermore, HS exposure downregulated the duodenal mucosal expression levels of antioxidant enzyme genes and *Nrf2*. Because of the antioxidant effect of natural polysaccharides in poultry [19,30,31,48], the alleviation effects of dietary supplementation of ADP on oxidative stress of the duodenal mucosa in broilers under HS should be explored. The results showed that dietary ADP supplementation improved the antioxidant capacity in the duodenum of broilers under HS, which was connected with the modulation of the Nrf2 signaling pathway. Similar to our study, heat-stressed broilers fed with natural polysaccharides (mannan oligosaccharides) had improved intestinal oxidative status [49]. Liu et al. [31] suggested that dietary ADP supplementation enhanced the intestinal antioxidant capacity of broilers. The enhanced antioxidant capacity in the duodenum by ADP supplementation could explain the improvement of tight junctions in this study.

### 4.4. Immune Response in Duodenum

It has been reported that HS causes a gut inflammation response in broilers [13], and NF-κB has an activation effect on the inflammation response in broilers under HS [50]. The NF-κB p65 is responsible for controlling the transcription of some specific genes, especially the inflammatory cytokines. Under normal conditions, NF-κB p65 is bound with IκBα and present in the cytoplasm inactively. When heat and cellular stress occur, IκBα is degraded, NF-κB p65 is activated immediately and then enters into the nucleus to regulate expressions of downstream inflammatory cytokines [11,44,51]. TNF-α and IL-1β are both secreted by activated macrophages, which have been considered proinflammatory cytokines [52], and the increased expression of the proinflammatory cytokines is another important mechanism of intestinal mucosal injury [53]. In this study, HS exposure downregulated *IκBα* expression and elevated *NF-κB p65*, *TNF-α* and *IL-1β* expression of duodenal mucosa. Suggesting that HS triggered the degradation of IκBα and the activation of NF-κB p65, thus promoting the transcription of proinflammatory cytokines in the duodenum. Similar results were found by Wu et al. [53] and Liu et al. [54]. The ADP has been suggested to have immunomodulatory properties [27,55,56], and the current study showed that the addition of ADP ameliorated the elevation of proinflammatory cytokine (*TNF-α* and *IL-1β*) expression and inhibited IκBα degradation and NF-κB p65 activation. Supporting findings were reported by Zhang et al. [57], who found that dietary ADP could improve the immune response of heat-stressed broilers. Previous studies also demonstrated that natural dietary polysaccharides decreased proinflammatory cytokine expression and suppressed IκBα/NF-κB p65 signaling activation in heat-stressed broilers [33] and model animals [58]. In the present study, the relieved duodenal inflammation response by supplementation of ADP indicated that there was a decreasing risk of inflammatory bowel disease in broilers exposed to HS, which also contributed to the enhancement of duodenal tight junctions.

## 5. Conclusions

In conclusion, the current results demonstrated the beneficial roles of ADP in improving duodenal tight junctions in heat-stressed, yellow-feathered broilers, and dietary supplementation of ADP could ameliorate HS-induced oxidative stress and the inflammation response in the duodenal mucosa via modulating Nrf2 and NF-κB signaling pathways. These findings indicate that dietary ADP supplementation is an effective nutritional approach to mitigate the detrimental effects of HS on gut health in broilers.

## Figures and Tables

**Table 1:** Influence of algae-derived polysaccharide (ADP) supplementation on duodenal antioxidant capacity in heat-stressed broilers.

Items	TN	HS	HSA	SEM	*p*-Value
T-AOC, mmol/mg protein	88.32 ^a^	47.81 ^b^	69.26 ^c^	6.25	0.004
T-SOD, U/mg protein	282.35	285.18	273.19	8.78	0.619
GSH-Px, U/mg protein	70.23	59.76	69.35	4.79	0.228
CAT, U/mg protein	4.15	3.09	2.99	0.44	0.178
GST, U/mg protein	27.46 ^a^	17.88 ^b^	25.87 ^a^	1.38	0.001
MDA, nmol/mg protein	1.37 ^a^	2.28 ^b^	1.55 ^a^	0.22	0.033

Note: TN group (thermoneutral zone, broilers exposed to 23.6 ± 1.8 °C); HS group (heat stress, broilers exposed to 33.2 ± 1.5 °C for 10 h/day); HSA, heat-stressed broilers were fed with ADP at 1000 mg/kg. SEM, standard error of mean. ^a,b,c^ The significant differences between groups were expressed as different superscript letters in the same row (*p* < 0.05).

## Data Availability

All datasets generated for this study are included in the article.
